# Chemical Composition and Biological Activities of Oregano Essential Oil and Its Fractions Obtained by Vacuum Distillation

**DOI:** 10.3390/molecules24101904

**Published:** 2019-05-17

**Authors:** Magdalena de J. Rostro-Alanis, Juan Báez-González, Cynthia Torres-Alvarez, Roberto Parra-Saldívar, José Rodriguez-Rodriguez, Sandra Castillo

**Affiliations:** 1 Tecnologico de Monterrey, Escuela de Ingeniería y Ciencias, Ave. Eugenio Garza Sada 2501, Monterrey 64849, N.L. México; magda.rostro@tec.mx (M.d.J.R.-A.); r.parra@tec.mx (R.P.-S.); 2 Facultad de Ciencias Biológicas, UANL, Av. Universidad s/n, Cd. Universitaria, San Nicolás de los Garza, Nuevo León 66455, México; juan.baezgn@uanl.edu.mx (J.B.-G.); cta_83@hotmail.com (C.T.-A.)

**Keywords:** oregano, *Poliomintha longiflora*, essential oil, antioxidant activity, antimicrobial activity, vacuum fractional distillation

## Abstract

Oregano (*Poliomintha longiflora)* essential oil (Ooil) is a product of high commercial value and many applications, including chemotherapy. Aiming to achieve the best use of this resource, the present study focuses on the characterization of separated fractions of Ooil by fractional vacuum distillation at low pressure. Four fractions (F1–F4) and undistilled oil (Unoil) were separated from Ooil and analyzed for their chemical composition and biological activities, such as antioxidant and antimicrobial activities. Gas chromatography–mass spectrometry shows differences in the composition among the fractions and Ooil. The amount of monoterpenes oxygenated (MO), sesquiterpenes hydrocarbon (SeH) and monoterpenes hydrocarbon (MH) varied between the fractions in ranges of 1.51–68.08, 3.31–25.12 and 1.91–97.75%, respectively. The major concentrations of MO and SeH were observed in F4 and Unoil. On the other hand, the highest concentrations of MH were found in F1 and F2, while the lowest were in F4 and Unoil. These results were correlated with the biological activity. Free-radical scavenging activity varied among fractions, with F4 and Unoil showing the highest activity. The antimicrobial test showed that F4 and Unoil had the highest activity in almost all cases. The correlation between the variables studied in the different fractions allows the definition of the particular properties for each one of them.

## 1. Introduction

Essential oils are “volatile oils or essences derived from vegetation and characterized by distinctive odors and a substantial measure of resistance to hydrolysis” according to the Encyclopedic Dictionary of Polymers [1,2]. These are a complex mixture of different volatile compounds present in aromatic plants in a natural way that, due to their properties and their fragrance, are widely used in cosmetics, in the food industry to improve the taste and the organoleptic properties, and in a variety of household products. In addition to their flavor and fragrance, many essential oils and their isolated components exhibit muscle-relaxing, antibacterial and antifungal activities. These properties are used in applications such as the preservation of raw and processed foods, pharmaceutical products and alternative medicine [2,3,4].

In recent years, due to restrictions on the use of synthetic food additives and the constant increase in the survival capacity of detrimental microorganisms caused by resistance to antibiotics and preservatives, there has been an increase in the search for alternatives, such as natural antimicrobial compounds [4,5]. In this sense, the effectiveness of the antimicrobial action of oils and extracts depends to a large extent on the chemical composition, which is related to the species and the parts of the plant that are used, the growing conditions, the harvest and the processing, as well as the method to obtain the extracts [2,5,6,7,8,9].

By chemical composition, essential oils are mostly terpenoids, including monoterpenes, sesquiterpenes and their oxygenated derivatives [4]. The qualitative-quantitative composition of several chemical compounds found in essential oils is important, since it is responsible for their antimicrobial efficacy [5]. That is, not only which compounds are present, but their proportion and amounts are important for their activity. A large number of aromatic plant species belong to the Lamiaceae family. Within this family, oregano (*Poliomintha longiflora)* is one of the most used aromatic plants, whose essential oils are particularly rich in mono- and sesquiterpenes [6]. 

In recent years, many of its therapeutic properties have been demonstrated: antioxidant [7,10,11], antimicrobial [4,10,12], anti-inflammatory [10,13,14], antiviral [11], antispasmodic [15], antiproliferative [4,16], and neuroprotective [17], among others. Therefore, the interest in this plant is increasing, especially as an important therapeutic alternative. The knowledge about oregano and the different processing methods is important for obtaining natural products of good quality, rich in active principles, with a well-determined composition, that can be recommended for different diseases and in efficient doses [10].

Considering the demand for oregano oil in the food, cosmetic and pharmaceutical industries, the focus of this work is the fractionation of oregano essential oil by fractional vacuum distillation at low pressure. The process will facilitate the physicochemical characterization and evaluation of the biological activity of each of the fractions obtained at different times. By means of the multivariate analysis of main components, the correlations of the different chemical compounds are established with the corresponding antimicrobial and antioxidant activities and these, in turn, with each of the fractions obtained.

This study intends to locate, by its composition and biological activity, each of the fractions available in the market for a particular purpose. The degree of correlation between the variables studied will define the particular purpose for each of the fractions, as well as their commercial value, for an integral use of the resource.

## 2. Results

### 2.1. Obtention of the Oregano Essential Oil and Fractions

**Figure 1:**
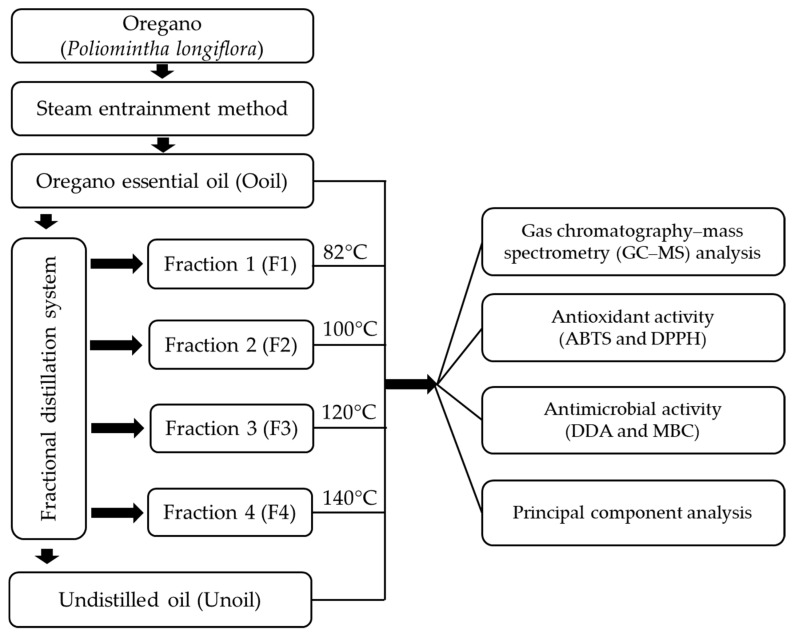
Diagram of the process for obtaining oregano essential oil and the oil fractions.

The diagram of the process of obtaining oregano essential oil (Ooil) through the steam entrainment method and the oil fractions through a fractional distillation system is shown in Figure 1. The first fraction started to distill at a temperature of 82 °C and the last fraction distilling at 140 °C, finally undistilled oil was obtained. At the end of the process, five fractions named Fraction 1 (F1), Fraction 2 (F2), Fraction 3 (F3), Fraction 4 (F4), and undistilled oil (Unoil) were obtained.

### 2.2. Physicochemical Characteristics

To determine differences in the physicochemical characteristics of each oil, analyses of brix degree, color, specific gravity and refractive index were performed. Table 1 shows the physicochemical characteristics of the Ooil, its fractions, and Unoil. Apart from the color, there are no great differences between the fractions, Unoil, and Ooil.

### 2.3. Gas Chromatography–Mass Spectrometry (GC–MS) Analysis

Each of the fractions obtained from the fractional distillation under vacuum were analyzed by GC-MS. Table 2 shows the profile of organic compounds found in the fractions analyzed, these are classified as monoterpene hydrocarbons (MH), monoterpenes oxygenated (MO) and sesquiterpene hydrocarbons (SeH). As shown, there is a clear difference in the type and quantities of the organic compounds present among the fractions, Unoil and Ooil. The amount of MO, SeH and MH varied between the fractions in ranges of 1.51–68.08, 3.31–25.12, and 1.91–97.75%, respectively. 

The compounds identified in the oregano essential oil are in a volatility range of 100–325 ° C and are classified as semivolatiles. The presence or absence of these compounds in the fractions is determined by the time and temperature range of the process according to the scheme of Figure 1. The most volatile terpenes in this group of compounds are present in F1 and F2, while the less volatile are present in F3 and F4. Some compounds, such as *o*-cymene and γ-terpinene, are present in different amounts in F1 to F3 and are almost insignificant in F4 and Unoil. These compounds were distilled little by little during the process. Less volatile terpenes such as carvacrol, *trans*-caryophyllene and α-humulene were detected in Unoil. Ooil contains all the terpenes found in the fractions and Unoil, but in different proportions. It is worth noting the absence of oxidized compounds, such as aldehydes and ketones, which indicates a self-protection of the oil to oxidation [2]. 

### 2.4. Antioxidant Activity

Several studies have analyzed the antioxidant activity of these compounds by various methods, such as 2,2′-azinobis-3-ethylbenzthiazoline-6-sulphonate (ABTS), 1,1-diphenyl-2-picrylhydrazyl (DPPH), ferric reducing antioxidant power (FRAP), the oxygen radical absorption capacity (ORAC), etc. [12,18,19]. The results may depend on the method used, and for that reason it is important to use at least two methods. The antioxidant activity values obtained for F1–F4, Unoil and Ooil were evaluated by the methods of ABTS radical and DPPH radical. The results are shown in Table 3. The values are expressed as [μmol TE/g evaluated oil (EO)]. The values obtained by the ABTS method ranged from 10.76–177,016.33 μmol TE/g EO, while the DPPH method showed values between 2.91 and 22,129.54 μmol TE/g EO. The F4 fraction showed the highest concentration of equivalent Trolox, with 177,016.31 μmol TE/g EO in ABTS followed by Unoil and Ooil (150,310.58 and 61,500.67 μmol TE/g EO respectively). In contrast, using the DPPH method, Unoil showed the best activity with 22,129.52 μmol TE/g EO, followed by F4 and Ooil (6025.03 and 4177.52 μmol TE/g EO, respectively). F3 showed considerable less activity using ABTS, compared with DPPH, with 26,002.33 and 161.83 μmol TE/g EO, respectively. Finally, F1 and F2 showed the lowest activity for the two methods evaluated. 

The half maximal inhibitory concentration (IC_50_) values are shown in Table 4, where F3, F4 and Unoil did not show significant differences to inhibit 50% of ABTS and DPPH radicals compared with Ooil. F1 and F2 showed significant differences for DPPH (114,507 mg/mL and 103,563 mg/mL) and ABTS (106,621 mg/mL and 160,796 mg/mL). These two fractions showed the least inhibition potential.

The results obtained in this work indicate that F4 and Unoil showed better antioxidant activity than the other oregano oils [2,10]. Previous studies have demonstrated that the components that promise more biological activity benefits are thymol and carvacrol. According to the chemical profile, these two compounds are present in larger percentages (4 to 5 times) in the F4 and Unoil fractions, compared with Ooil, while they are absent in F1 and F2. In the case of F3, thymol is absent but carvacrol is present. This fraction (F3) presented low antioxidant activity compared with F4 and Unoil, but better than F1 and F2. These results can be attributed to the fact that antioxidant activity is correlated with the type and amount of compounds presented. Despite the lack of thymol and low amount of carvacrol in F3, its biological activity could be enhanced by synergistic effect with the other compounds present [20]. Other studies have established that the essential oregano oil has good antioxidant activity using DPPH and ABTS. The components mainly responsible for this activity are carvacrol and thymol (both oxygenated monoterpenes), attributed to the presence of hydroxyl groups in phenolic compounds. It is important to consider that the potential antioxidant activity of oregano oil may also be due to a possible synergy between other oil compounds [21]. F4 and Unoil showed greater antioxidant activity than F1, F2, F3, and even oregano essential oil. This greater activity could be explained, in part, by the chemical profile of F4 and Unoil, which have a higher content of compounds such as carvacrol and thymol, the latter compound even by as much as 50% as compared with other fractions. This trend was also observed by Olmedo et al. (2014), who investigated the antioxidant activity of distilled oregano oil and residues. They found that the distillates and residues, with higher phenolic content showed greater antioxidant activity. It is known that phenolics are hydrogen donors to other molecules, which increases their antioxidant activity [7].

### 2.5. Antimicrobial Activity

The antimicrobial activity was evaluated through preliminary assays of disk diffusion test and the minimum inhibitory concentration (MIC) assessment. The antimicrobial activity was tested against pathogens of both food and clinical importance. Representative groups of Gram-positive bacteria (*L. monocytogenes* and *Staphylococcus aureus*), Gram-negative (*Salmonella typhi*) and a fungal species (*Candida albicans*) were selected due to their physiological differences. The results of the preliminary assay (disk diffusion) are shown in Table 5. These results demonstrate antimicrobial activity for the Fractions 3, 4, and Unoil, with inhibition zones ranging from 1–4.5 cm. There was no significant difference between the activity presented by the F3 fraction and control (Ooil), while F1 and F2, did not show any antimicrobial activity. On the other hand, the Unoil and F4 showed the best activity in all cases compared with control (Ooil). This can be attributed to the presence of non-volatile compounds (MO2, MO3, SeH1, SeH2) that are found in greater quantities in Unoil and F4 compared with fractions 1 and 2, in which those compounds are absent and instead possess volatile compounds (MH). Previous studies have demonstrated that volatile compounds (MH) are unstable and when such compounds are removed, others of more interest, industrially or biologically, are enriched [20]. Fraction 3 contains only carvacrol, but not thymol. Carvacrol accounts for the main antimicrobial activity and could work in synergy with other compounds to enhance antimicrobial activity [20]. Furthermore, some studies recognize that carvacrol and γ-terpinene are responsible in considerable degree for the antimicrobial activity [21]. Both compounds are present in F3, while γ-terpinene is nearly twice as abundant as in Ooil (Table 2).

The results of the evaluation of MIC are shown in Table 6. In these results, the same pattern was observed for *S. typhi* and *C. albicans*, with Unoil and F4 showing the best antimicrobial activity. On the other hand, when tested against *S. aureus* and *L. monocytogenes*, F3 and F4, showed less activity compared with Unoil and Ooil. In this particular case, it is important to consider the decrease of carvacrol in both fractions (F3, F4) compared to Unoil because it is the main component to which the antimicrobial activity is attributed. On the other hand, despite the Ooil containing lower amount of carvacrol than F4 it contains a great variety of components that can act synergistically [20]. These results varied respect to the disk diffusion assay, perhaps because the size of the inhibition zone can be influenced by chemical composition of the essential oil, rate of diffusion into the agar, and volatility which may have affected the MIC [22]. In addition, antimicrobial activity is not attributable to one specific mechanism, therefore could vary from one species to another, due to the physiological differences between them [21]. 

### 2.6. Analysis of Main Components

**Figure 2:**
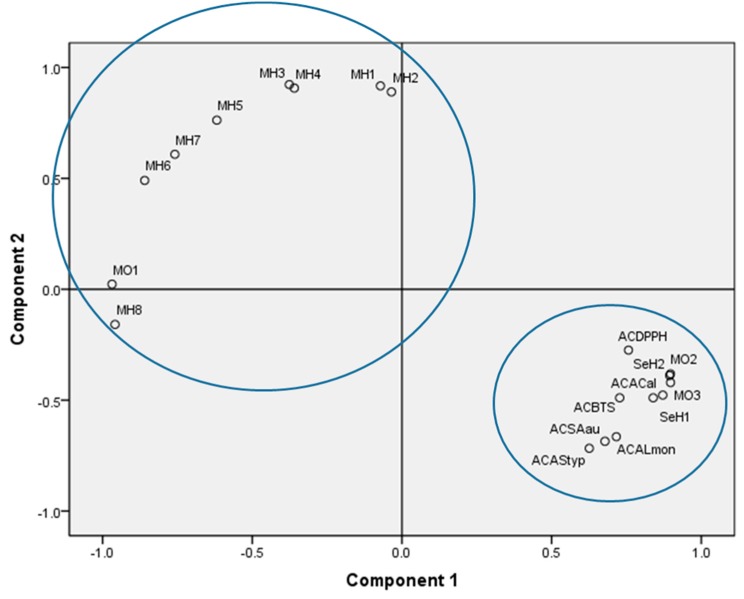
Diagram of the variables generated by the analysis of main components. ACDPPH means antioxidant activity by DPPH Methods, ACBTS means antioxidant activity by ABTS method, ACSAau means antimicrobial activity against *Staphylococcus aureus*, ACALmon means antimicrobial activity against *Listeria monocytogenes*, ACAStyp means antimicrobial activity against *Salmonella typhi*, ACAcal means antimicrobial activity against *Candida albicans*.

After determining the components and biological activity of each oil, an analysis of main components was performed. The components used in this work, according to the extraction method of the analysis of main components, were component 1 and component 2. These two components account for 91% of the variation in the multivariate analysis (Table S2). As shown in Figure 2, the variation provided by the measured parameters can be explained with two groups: Group 1, that mainly groups together non-volatile compounds which strongly correlate with the determined biological activities; and Group 2, that groups volatile organic compounds which do not have a correlation with the biological activities evaluated in this work. In addition, as can be observed, the variables MO1 and MH8 have a strong correlation with the group of volatile organic compounds. 

**Figure 3:**
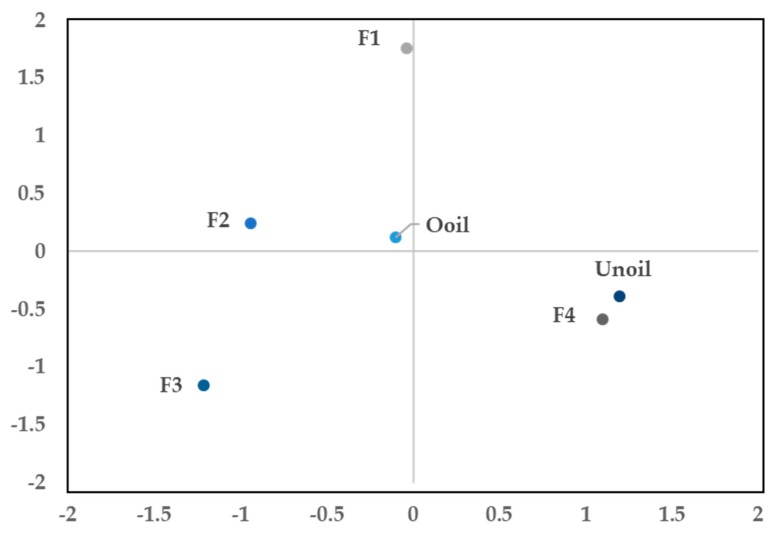
Diagram of the fractions in a single factorial plane.

Figure 3 shows the diagram of the fractions in a single factorial plane, where the coordinates agree with the graph of main components. In this way it can be observed that F4 and Unoil contain the compounds which can be attributed to the antimicrobial and antioxidant activities, unlike the F1 and F2 fractions, that clearly are characterized as containing the volatile compounds. F3 stands out because it contains aromatic compounds, antioxidant and antimicrobial activities, although the latter are smaller than F4 and Unoil. Given the location of the Ooil, in the center of all the fractions, this should be considered with a chemical composition and biological activities lower than the corresponding fractions derived from it.

## 3. Discussion

The constituents and the concentration of essential oil compounds found in oregano generally vary due to a wide diversity of factors such as species, pests, soil conditions, harvest season, geographical location, climate, and growing conditions [2,5]. In addition, the drying method, the extraction technique and the anatomical part of the plant used for the extraction also influence the yield and composition of the essential oils [7,8,9]. Our work shows that an essential oil fractionated under different temperature and vacuum conditions contains a different profile of chemical compounds that directly influence the biological activities presented. 

In our study, we determined the quantitative and qualitative composition of the bioactive compounds, the predominant compounds in each of the fractions and the antioxidant and antimicrobial activities in each of them. The chemical composition of oregano (Ooil), of its fractions obtained by fractional vacuum distillation (F1–F4) and the undistilled remnant (Unoil) was different in each of them. 

α-thujene, α-pinene, myrcene, α-terpinene, *p*-cymene, β-phellandrene, limonene, and γ-terpinene were found in greater proportion in F1 and F2, this means that are considered more volatile. This coincides with the report by Borgarello et al. and Olmedo et al. [7,9]. According to the analysis of main components performed in this study, the F1 and F2 fractions have no correlation with the biological activities evaluated. 

On the other hand, the MO compounds (thymol and carvacrol), and SeH (*trans*-caryophyllene and α-humulene) were not detected in F1 and F2, while they were present in a larger proportion in F4 and Unoil, and in smaller concentration, in Ooil. According to the principal component analysis, these last components are correlated with the biological activity. In addition, it was found that Unoil and F4 had greater biological activity than Ooil in most cases due to the larger concentration of these last compounds and the smaller amounts of other volatile compounds, indicating a possible synergy between the remaining constituents of these fractions [23,24]. F1 and F2 contained no SeH nor MO, which clearly are responsible for the biological activity; therefore, these two fractions had low or no activity. Other studies have confirmed that these compounds confer biological properties [2,6,10,25,26,27], and it has been suggested that they may have synergy with minor oxygenated components [26]. Using the multivariate analysis in this study, specifically the PCA, the correlation between the antioxidant and antimicrobial activities with the phenolic monoterpenes: thymol, carvacrol, and sesquiterpenes: *trans*-caryophyllene, α-humulene, is evident.

Borgarello et al. (2015) obtained similar results when studying different conditions by molecular distillation, obtaining fractions and distillates, finding that the fractions showed an increase in the antioxidant activity while the distillates showed a decrease in this property, perhaps due to the presence of minor oxygenated compounds in synergy or antagonism [9]. In addition, Olmedo et al. (2014) mentioned that different chemical compositions of oregano essential oils produced different antioxidant activity [7]. For that reason, increasing the concentration of a particular molecule with high antioxidant potential in a fraction obtained from the essential oil should increase the antioxidant power in that fraction, compared with the antioxidant activity of the essential oil. 

With respect to the antimicrobial activity, F3, F4, Ooil, and Unoil were effective in inhibiting microbial growth and showed zones of inhibition diameters comparable with other publications [2,4,5,8,10]. The results of MIC are consistent with those obtained by the diffusion method.

F3, F4, Ooil, and Unoil worked as inhibitors against all the bacteria analyzed and all of them contain MO and SeH compounds. Within the mechanisms of action of the antimicrobial activity of essential oils, it has been reported that the hydrophobicity of the molecules or even the position of the hydroxyl groups have an important role in the penetration through the membrane. F4 and Unoil contain major quantities of carvacrol and thymol; these two compounds have a prominent disintegrating effect on the outer membrane; this could explain the major antimicrobial activity exerted by these two fractions. Even though the Gram-positive bacteria generally are more sensitive than Gram-negative, the mechanism of action against them differ due to the different structures of the cell walls and the response could vary. Previous studies have demonstrated that some essential oils such as rosemary and oregano are active against *E. coli* (Gram-negative) and *Bacillus cereus* (Gram-positive) but less effective for *Pseudomonas* spp. (Gram-negative) Additionally, it has been proven that different strains of the same bacteria can behave differently when exposed to the same essential oil or their components [28]. There is some conflict between the results of some investigations. In some cases the antimicrobial activity of a single compound has been demonstrated (e.g., γ-terpinene, *p*-cymene) [21], while other studies have concluded that there is no such activity [28]. Compounds such as carvacrol and thymol have been widely studied. MICs have been reported for: *S. typhimurium* (312,375 μg/mL) [29], *L. monocytogenes* (150,250 μg/mL) [30], *S. aureus* (200,200 μg/mL) [31] and *C. albicans* (256,390 μg/mL) [32], respectively. According to the results obtained in this study, and compared with those reported in the literature, Unoil and F4 continue to show greater antimicrobial activity in almost all cases. Even Ooil showed greater antimicrobial activity for *L. monocytogenes* and *C. albicans* compared with the individual compounds reported in the literature. This agrees with previous studies where it has been shown that individual compounds, in some cases, have little antimicrobial activity. Such is the case of *p*-cymene and γ-terpinene, which have little antimicrobial activity when tested individually, but are capable of potentiating the effect of compounds such as carvacrol [33]. In addition, there is a synergy between carvacrol and thymol, which show better activity when they are together [34]. On the other hand, it has been demonstrated that α-humulene has a good antimicrobial activity with MIC’s reported of 3.90 and 2.6 μg/mL for *E. coli* and *S. aureus* respectively [35,36]; Unoil and F4 have greater amount of α-humulene than Ooil, which could explain the major antimicrobial activity of these two fractions compared to the others, where neither α-humulene, nor carvacrol and thymol. Although hypotheses of the action mechanisms of essential oils have been described, more research is needed to fully clarify the mode of action of the antimicrobial activity [28].

Our results show a good antimicrobial activity (inhibition diameter between 1.0 and 2.4 cm), comparable, or better, than those obtained by other authors who indicated a moderate to good antibacterial activity of the oregano extract. It should be noted that our results are also comparable to those reported for gentamicin (1.8 to 2.2 cm) used as a standard antibacterial agent [10].

Furthermore, the results obtained against *C. albicans* indicate that the F4, Ooil and Unoil, have a good antifungal effect (diameter of inhibition of 4.3–4.5 cm) compared to that reported with the antifungal drug Amphotericin B (2.1 cm in diameter) [10]. The origin of the plant material, as well as the method of obtaining the essential oil fractions, were determinants for the antifungal activity, according to reports by García and collaborators who evaluated oregano oil extracted by supercritical fluids against the same strain of *C. albicans* and obtained less than 1 cm of inhibition [8]. 

Our results showed an increment of certain compounds due to the extraction by vacuum distillation that enhanced the antioxidant and antimicrobial activity.

## 4. Materials and Methods

### 4.1. Plant Material and Reagents

The plant material of oregano (*Poliomintha longiflora*) was collected in a region of the sierra where the Mexican states of Coahuila, Durango, and Zacatecas converge. The coordinates are 24°55” latitude (north) and 103°10” longitude (west). The plant was collected 15 days after the rain in May 2018. The phenological stage of the plant at the time of harvest was during and 10 days after flowering. The composition of the vegetal material used was leaf, flower, and stem in a proportion of 90:9:1, respectively. The specimen was identified and deposited in the herbarium of the Facultad de Ciencias Biológicas, UANL and the corresponding coupon was generated.

The reagents used were analytical grade: 2,2′-azinobis-3-ethylbenzthiazoline-6-sulphonate (ABTS) radical, 1,1-diphenyl-2-picrylhydrazyl (DPPH) radical, and Trolox. They were purchased from Sigma-Aldrich (Sigma chemical Co., St. Louis, MO, USA). 

### 4.2. Acquisition of the Oregano Essential Oil and Fractions

Oregano essential oil was obtained through the steam entrainment method provided by the Or-Lag company. The fractions of oil were obtained through a fractional distillation system with a distillation column equivalent to 20 theoretical plates at a pressure of 5 Torr from Frutech International (Guadalupe, NL, Mexico). The distillation temperature range was 82 to 140 °C. The collection period between each fraction was approximately 20 min. 

### 4.3. Physicochemical Characteristics of the Oils

The physicochemical characteristics of each oil were determined using different tecniques. Analysis of brix degree, color, specific gravity and refractive index were performed acording to Torres-Alvarez et al. [20]. 

### 4.4. Gas Chromatography–Mass Spectrometry (GC–MS) Analysis

The analysis of the Ooil, its fractions (F1–F4) and Unoil, was performed under the following conditions: GC–MS (6890/5973N), Agilent Technologies (Santa Clara, CA, USA). GC was conducted on a HP-5 MS (30 m × 0.25 mm × 0.25 μm) capillary column. The GC conditions were as follows: injection temperature 250 °C; the oven temperature was controlled at 70 °C for 1 min with the heating rate from 10 °C/min to 200 °C for 2 min, and finally from 10°C/min to 300 °C for 5 min. Helium gas was used as carrier gas at a constant flow rate of 1 mL/min, sample size at 1 μL. The parameters for MS analysis 5973N were with EI ion source, electron energy 70 eV, the temperature of quadrupoles 150 °C, temperature of interface 230 °C, m/z = 30–400 amu. Identification of compounds was carried out by comparing their mass spectra with those of Wiley 7 n.L library, considering a quality match >85%. Additionally, the identification was carried out using the index of Kovats (IK) obtained and compared with the values published in literature (Table S1).

### 4.5. Antioxidant Activity

The antioxidant activity of the oils was measured using the ABTS radical and DPPH radical. Trolox (Sigma–Aldrich, St. Louis, MO, USA), a water-soluble analogue of vitamin E, showing a potent antioxidant activity, was used as a standard reference (positive control). The ABTS radical scavenging assay was determined following the method according to Torres-Alvarez [20], with some modifications, the blue-green ABTS radical cation chromophore (ABTS·+) was prepared by reacting ABTS stock solution (7 mmol) with potassium persulphate (2.45 mmol) and allowing the mixture to stand at room temperature in the dark for 16 h. The solution was diluted with ethanol to obtain an absorbance of 0.700 ± 0.02 at 734 nm in a Genesys 5 (spectronic) spectrophotometer. The DPPH radical scavenging assay was conducted according to Torres-Alvarez [20]. The DPPH solution was prepared with 3.9 mg of radical and 100 mL of ethanol, and the solution was measured to 517 nm in a spectrophotometer to obtain an absorbance of 1.000 ± 0.05. Aliquots of 10–300 μL of solution of each oil with ethanol were mixed with 2.7 mL of ABTS·+ solution. The mixture was allowed to react at room temperature for 7 min in dark conditions. The absorbance was recorded using a spectrophotometer at 734 nm. For the DPPH assay, different concentrations (200–600 μL) of each oil were added separately to 2.25-mL DPPH solution. The mixture was shaken and left at room temperature for 240 min in the dark. Thereafter, the absorbance was measured at 517 nm in a spectrophotometer. The antioxidant activity was calculated as a percentage of inhibition according to the following equation:

%Inhibition = {[Ab − As/Ab] × 100},(1)

where Ab represents the absorbance of the control (without test oil), and As represents the absorbance of the test oil. A calibration curve was determined for the Trolox standard for each radical (ABTS and DPPH) at concentrations in the range of 10–160 μmol. Triplicate analyses of each oil were made. The antioxidant activity values were expressed as mmol Trolox equivalent (TE)/mL of the evaluated oil. In addition, the antioxidant activity expressed as half-maximal inhibitory concentration IC_50_ (mg/mL) was defined as the amount of antioxidant necessary to decrease the initial ABTS and DPPH concentration by 50% [37]. 

### 4.6. Antimicrobial Activity

#### 4.6.1. Microbial Strains and Growth Conditions

Three bacterial strains, *Salmonella typhi* (ATCC 19430), *Staphylococcus aureus* (ATCC 6538), and *Listeria monocytogenes* (ATCC 7644), were used in the study and kindly provided by the Laboratory of Sanitary Microbiology, FCB, UANL. The bacterial strains were stored at −80 °C in brain heart infusion (BHI; Difco Laboratories, Sparks, MD, USA) with 20% (*v*/*v*) glycerol. The fungal species Candida albicans ATCC 10231 (provided by Tecnologico de Monterrey) was stored under the same conditions but in potato dextrose broth (PDB; Difco Laboratories, Sparks, MD, USA) instead of BHI. Fresh cultures of microorganisms were subcultured in Mueller Hinton broth or agar (MHB and MHA; Difco Laboratories, Sparks, MD, USA). *L. monocytogenes* was cultivated on trypticase soy broth or agar (TSB, TSA; Difco Laboratories, Sparks, MD, USA) and *C. albicans* on PDB or potato dextrose agar (PDA). Briefly, an aliquot (50 μL) was taken from a frozen culture and was added to a tube containing 5 mL of MHB, TSB or PDB, depending of each strain, and were incubated at 37 °C for 18 h (30 °C for *L. monocytogenes*); After incubation, an aliquot (10 μL) of these fresh cultures was used for further assays.

#### 4.6.2. Preliminary Antimicrobial Activity

A preliminary antimicrobial activity of the Ooil, its fractions (F1–F4) and Unoil, was assessed against four pathogens, using the disk diffusion technique [20]. An aliquot (100 μL) of fresh culture suspension (adjusted to 0.5 McFarland ≈ 108) was distributed homogeneously on MHA, TSB, or PDA plates. Filter paper disks (Whatman No. 1) of 6 mm in diameter were separately impregnated with 10 μL of each oregano oil fraction and placed on the agar surface. The plates were incubated at 37 °C for 24 h. The antimicrobial activity was determined by the absence of bacterial growth in the area surrounding the disk. The diameter of the inhibition zones was measured with a caliper. The tests for *L. monocytogenes* and *C. albicans* carried out in the same way mentioned above, but TSA and PDA were used respectively instead of MHA; *L. monocytogenes* was incubated at 30 °C. Duplicate analyses were performed at least three times. Disks impregnated with sterile distilled water and/or DMSO served as negative controls while Ooil (whole oil) served as positive control.

#### 4.6.3. Assessment of Minimum Inhibitory Concentration

After determining the preliminary antimicrobial activity, the MIC values were obtained through the dilution method with minor modifications [20,38]. Briefly, an aliquot (50 μL) of fresh cultures adjusted to McFarland standard (0.5 ≈ 1 × 10^8^) of each strain were added separately in tubes containing 5 mL of MHB, TSB or PDB with different concentrations of the samples to evaluate (F1, F2, F3, F4, Unoil, and Ooil) in DMSO (Sigma), to give final concentrations ranging between 50 and 2000 µg/mL (final concentration in each tube for every oil). The cultures were then incubated at 37 °C for 24 h (30 °C for L. monocytogenes). After that, bacterial survival was determined by dropping 20 µl of each culture onto agar plates and incubated as described above. MIC was defined as the lowest concentration of oregano fraction oil that showed no bacterial growth on agar plates. Analyses were conducted by triplicate at least three times. Controls and blank samples were incubated under the same conditions. The final concentration of DMSO did not exceed 2% *v*/*v* and did not affect the bacterial growth [27].

### 4.7. Principal Component analysis

An analysis of the main components (PCA) was performed to find out whether there was a correlation between the variables, in order to find the minimum number of factors or components that can explain the maximum percentage of information provided by the variables involved in this analysis. The variables were: % of relative areas for each compound, antioxidant activity (μmol TE/mL for both methods DPPH and ABTS) and antimicrobial activity for each strain according to the method of inhibition zone reported for each of the oils under study (F1, F2, F3, F4, Unoil, and Ooil).

### 4.8. Statistical Analysis

All experiments were performed in duplicate at least three times. Statistical analyses were performed using SPSS software (version 10.0, SPSS Inc., Chicago, IL, USA) analyzed with an analysis of variance test; a post hoc test of Tukey´s HSD was used for the analyses of mean comparison. Differences between means were considered significant at *p*-alues of ≤ 0.05. The multivariate analysis of Principal component analysis (PCA) was performed using SPSS (Version 19, IBM Corp, and Chicago, IL, USA).

## 5. Conclusions

The oregano essential oil, its fractions and the non-distilled fraction obtained by the fractional vacuum distillation process differ in terms of content and quantitative proportion of terpenes and terpenoids, as well as in their biological activity.

Our results show an increase in certain compounds due to the extraction method (by vacuum distillation) that led to an increase in biological activity; therefore, this method provides a useful alternative to obtaining fractions of essential oils. The biological activity is correlated with the amount of MO and SeH present. These compounds, even in low amounts, could work in synergy, and maintain biological activity.

These findings, considered together, represent an important result, with the view, in the future, of using essential oils for different applications. Furthermore, the concentrated compounds correlated with the biological activities present in fractions F3, F4, and Unoil and offer an equal or greater antimicrobial activity compared to the original oil. These fractions can be a viable alternative for the health sector as antimicrobials that counteract resistance to pathogenic strains.

The fractions F1 and F2 can be used for their antioxidant activity in applications, such as natural preservatives for food products that, due to their colorless and soft odor, can offer a high degree of acceptance by the consumer.

## Figures and Tables

**Table 1:** Physicochemical characteristics of the oil fraction.

Oil	Code	Color	Odor	Specific Gravity (20 °C) g/mL	Refractive Index (20 °C)	Brix
Fraction 1	F1	Colorless	Soft	0.842 ± 0.0	1.47 ± 0.0	75.72 ± 0.0
Fraction 2	F2	Colorless	Soft	0.845 ± 0.0	1.48 ± 0.0	77.20 ± 0.0
Fraction 3	F3	Colorless	Strong	0.859 ± 0.0	1.48 ± 0.0	77.77 ± 0.0
Fraction 4	F4	Colorless	Strong	0.884 ± 0.0	1.51 ± 0.0	88.95 ± 0.0
Undistilled oil	Unoil	Brown	Strong	0.927 ± 0.0	1.51 ± 0.0	89.16 ± 0.0
Oregano Oil	Ooil	Light Yellow	Strong	0.778 ± 0.0	1.48 ± 0.0	76.06 ± 0.0

**Table 2:** Profile of the organic compounds found in the fractions analyzed.

Compound	Boiling Point °C	Code	% de Relative Area ^1^					
			F1	F2	F3	F4	Unoil	Ooil
α-thujene	150–152	MH1	5.03	0.389	ND	ND	ND	1.74
α-pinene	156	MH2	3.01	ND	ND	ND	ND	1.07
β-myrcene	166–168	MH3	11.62	6.93	1.08	ND	ND	5.50
Phellandrene	172	MH4	1.32	1.00	ND	ND	ND	0.72
α-terpinene	174	MH5	8.91	8.32	2.90	ND	ND	5.57
*o*-cymene	174	MH6	47.96	53.97	38.14	1.31	0.973	39.13
Limonene	175	MH7	2.29	2.71	1.25	ND	ND	1.58
1,8-cineole	177	MO1	1.51	1.77	2.74	ND	ND	1.53
γ-terpinene	181–183	MH8	15.59	24.43	40.57	1.40	0.94	22.34
Thymol	232	MO2	ND	ND	ND	5.08	3.77	1.71
Carvacrol	237–238	MO3	ND	ND	4.58	60.03	64.31	12.60
*Trans*-caryophyllene	268	SeH1	ND	ND	2.97	18.96	13.78	3.47
α-humulene	276	SeH2	ND	ND	0.34	6.16	8.36	1.56
Monoterpene hydrocarbons (MH)			95.73	97.75	83.94	2.71	1.91	77.65
Monoterpene oxygenated (MO)			1.51	1.77	7.32	65.11	68.08	15.84
Sesquiterpene hydrocarbons (SeH)			ND	ND	3.31	25.12	22.14	5.03
Total identified components			97.24	99.52	94.57	92.94	92.13	98.52

^1^ Given as percentage of mean peak area from triplicate determination. ND: Not detected.

**Table 3:** Antioxidant activity (μmol TE/g evaluated oil).

Oil	Method	
	DPPH	ABTS
F1	2.91 ± 0.58 ^d^	14.07 ± 1.14 ^e^
F2	6.71 ± 1.10 ^d^	10.76 ± 2.91 ^e^
F3	161.83 ± 4.76 ^d^	26,002.33 ± 1220.15 ^d^
F4	6025.03 ± 230.78 ^b^	177,016.31 ± 7369.93 ^a^
Unoil	22,129.54 ± 615.53 ^a^	150,310.58 ± 3609.10 ^b^
Ooil	4177.52 ± 181.62 ^c^	61,500.67 ± 522.20 ^c^

Mean values of three replicates ± the standard deviation of the mean. ^a–d^ Mean values with different letter in the same column are significantly different (*p* ≤ 0.05) Tukey’s HSD test. ^a–e^ Mean values with different letter in the same column are significantly different (*p* ≤ 0.05) Tukey´s HSD test.

**Table 4:** Inhibition concentration (IC_50_) mg/mL.

Oil	Method	
	DPPH	ABTS
F1	114,507 ± 15,060 ^b^	106,621 ± 1454 ^b^
F2	103,563 ± 13,021 ^b^	160,796 ± 2297 ^c^
F3	3968 ± 271 ^a^	162 ± 10 ^a^
F4	563 ± 51 ^a^	17 ± 1 ^a^
Unoil	117 ± 17 ^a^	22 ± 2 ^a^
Ooil	886 ± 23 ^a^	85 ± 3 ^a^

Mean values of three replicates ± the standard deviation of the mean. ^a–d^ Mean values with different letter in the same column are significantly different (*p* ≤ 0.05) Tukey´s HSD test.

**Table 5:** Results of disk diffusion assay. Inhibition zones are given in centimeters (cm).

Oil Fraction	Diameter of Inhibition Zone (cm)			
	*Staphylococcus aureus*	*Listeria monocytogenes*	*Salmonella typhi*	*Candida albicans*
F1	WI	WI	WI	WI
F2	WI	WI	WI	WI
F3	1.0 ± 0.05 ^a^	1.2 ± 0.15 ^a^	1.2 ± 0.1 ^a^	1.4 ± 0.16 ^b^
F4	2.0 ± 0.2 ^c^	2.4 ± 0.26 ^b^	2.19 ± 0.2 ^c^	4.5 ± 0.05 ^a^
Unoil	1.6 ± 0.2 ^b^	2.3 ± 0.2 ^b^	1.6 ± 0.1 ^b^	4.4 ± 0.1 ^a^
Ooil	1.1 ± 0.06 ^a^	1.4 ± 0.05 ^a^	1.15 ± 0.1 ^a^	4.3 ± 0.2 ^a^
Gentamicin [10]	1.8 ± 0.00 ^bc^	2.2 ± 0.00 ^b^	2.8 ± 0.7 ^d^	-
Amphotericin B [10]		-	-	2.1 ± 0.00 ^c^

Zone of inhibition are average values of three replicates ± the standard deviation of the mean. ^a–d^ Mean values with different letter in the same column are significantly different (*p* ≤ 0.05) Tukey´s HSD test. WI means without inhibition.

**Table 6:** Minimum inhibitory concentration (μg/mL) of each fraction.

Oil Fraction	Minimum Inhibitory Concentration (μg/mL)			
	*Staphylococcus aureus*	*Listeria monocytogenes*	*Salmonella typhi*	*Candida albicans*
F1	ND	ND	ND	ND
F2	ND	ND	ND	ND
F3	482 ± 20 ^c^	162 ± 16 ^c^	480 ± 11 ^c^	450 ± 24 ^b^
F4	660 ± 54 ^b^	164 ± 19 ^c^	135 ± 13 ^b^	84 ± 10 ^a^
Unoil	276 ± 25 ^a^	6.4 ± 0.8 ^b^	115 ± 13 ^b^	78 ± 10 ^a^
Ooil	280 ± 23 ^a^	77 ± 0.7 ^a^	770 ± 27 ^a^	94 ± 5 ^a^
Gentamicin [10]	3.2 ± 0.5 ^d^	2.0 ± 0.3 ^b^	>122 ± 10 ^b^	
Amphotericin B [10]				0.25 ± 0.05 ^c^

Are average values of three replicates ± the standard deviation of the mean. ^a–d^ Mean values with different letter in the same column are significantly different (*p* ≤ 0.05) Tukey´s HSD test. ND means Not Detected.

## Supplementary Materials

The following are available online, Tables S1 and S2.
