# Supplementary material for: Chemical Composition and Biological Activities of Oregano Essential Oil and Its Fractions Obtained by Vacuum Distillation
Source: Molecules. 2019 May 17;24(10):1904. doi: 10.3390/molecules24101904 (PMC6571842; doi:10.3390/molecules24101904)
Supplement: Supplementary file 1 [file molecules-24-01904-s001.pdf]

Supplementary Material

Table 1S. Calculation of the Kovats index of the compounds identified in the GC-MS.

Compound	Retention time	Retention time of decane trx = 10	Retention time of dodecane trx = 12	Kovats index calculated
α-thujene	3.752	4.55	7.31	919
α-pinene	3.856	4.55	7.31	930
β-myrcene	4.488	4.55	7.31	994
Phellandrene	4.717	4.55	7.31	1015
α-terpinene	4.877	4.55	7.31	1029
o-cymene	4.985	4.55	7.31	1039
Limonene	5.042	4.55	7.31	1043
1,8-cineole	5.089	4.55	7.31	1047
γ-terpinene	5.444	4.55	7.31	1076
Thymol	8.661	4.55	7.31	1272
Carvacrol	8.804	4.55	7.31	1278
Trans-caryophyllene	10.514	4.55	7.31	1353
α-humelene	10.946	4.55	7.31	1370

Table 2S. Extraction method: Principal Components Analysis. Total variance explained.

Component	Initial eigenvalues			Sums of saturations to the square of the extraction			Sum of the saturations to the square of the rotation		
	Total	Percentage of variance	Percentage accumulated	Total	Percentage of variance	Percentage accumulated	Total	Percentage of variance	Percentage accumulated
1	14.72	77.45	77.45	14.72	77.45	77.45	10.17	53.53	53.53
2	2.66	14.01	91.46	2.66	14.01	91.46	7.21	37.93	91.46
3	0.82	4.31	95.77						
4	0.48	2.52	98.29						
5	0.33	1.72	100.00						
6	5.91×10^{-16}	3.11×10^{-15}	100.00						
7	4.08×10^{-16}	2.18×10^{-15}	100.00						
8	3.54×10^{-16}	1.86×10^{-15}	100.00						
9	2.58×10^{-16}	1.36×10^{-15}	100.00						
10	2.35×10^{-16}	1.24×10^{-15}	100.00						
11	1.91×10^{-16}	1.00×10^{-15}	100.00						
12	2.99×10^{-17}	1.57×10^{-16}	100.00						
13	-1.03×10^{-16}	-5.40×10^{-16}	100.00						
14	-1.65×10^{-16}	-8.68×10^{-16}	100.00						
15	-1.80×10^{-16}	-9.50×10^{-16}	100.00						
16	-2.54×10^{-16}	-1.34×10^{-15}	100.00						
17	-3.54×10^{-16}	-1.86×10^{-15}	100.00						
18	-4.13×10^{-16}	-2.17×10^{-15}	100.00						
19	-5.34×10^{-16}	-2.81×10^{-15}	100.00						
